# Blame it on my youth: the origins of attitudes towards immigration

**DOI:** 10.1057/s41269-023-00314-6

**Published:** 2023-10-28

**Authors:** Anne-Marie Jeannet, Lenka Dražanová

**Affiliations:** 1 University of Milan, Via Conservatorio 7, Milan, Italy; 2 European University Institute, Via delle Fontanelle 19, Fiesole, Italy

**Keywords:** Political socialization, Age-period-cohort analysis, Attitudes to immigration, Political climate

## Abstract

Recent studies have demonstrated generational differences in attitudes towards immigration, however, less is known about what are the exact factors behind these differences. Our study investigates why cohorts formulate distinct patterns in attitudes towards immigration through a collective process of political socialization during their formative years. The theoretical arguments are tested using hierarchical age-period-cohort modelling across thirteen cohorts in thirteen European countries using micro attitudinal data (2002–2020) integrated with historical macro-political data. We find that contextual exposure to the principle of equality in the formative political climate is central to the formulation of a person’s attitudes towards immigration later in life. While the prevalence of the principle of equality affects immigration attitudes in adulthood positively, the principle of tradition does not. The findings imply that even subtle and cyclical shifts in national politics affect the political orientations of those undergoing the process of political socialization.

**Supplementary Information:**

The online version contains supplementary material available at 10.1057/s41269-023-00314-6.

## Introduction

The issue of immigration divides generations, prompting scholarly discussions about how these differentiations emerge. Earlier studies have shown that older people are more likely to express concerns about immigration or hold negative attitudes about immigrants than younger people (Mayda 2006; Quillian 1995). Instead, some suggest that differences in attitudes across age groups are due to an ideological shift between younger and older generations (Wilkes and Corrigall-Brown 2011). Recent research has empirically demonstrated that age-specific patterns regarding immigration attitudes are due to a person’s year of birth, rather than his or her biological age (Calahorrano 2013; Gorodzeisky and Semyonov 2018; McLaren and Paterson 2019; Schotte and Winkler 2018; Ford 2011). The reasons for this are not immediately apparent, as the trend from one cohort to the next is non-linear and fluctuates (Gorodzeisky and Semyonov 2018). For example, Schmidt (2021) finds that while cohort replacement has led to a substantially more positive opinion toward immigrants since the 2000s, younger generations born between 1982 and 1991 are more concerned about immigration than the older ones. Moreover, patterns of attitudes towards immigration amongst young cohorts differ across country contexts (Munck et al. 2018). In other words, it is not simply a matter of older generations being more against immigration than younger generations. Instead, it appears that age cohorts, individuals born around the same time, experience a unique set of common circumstances constituting a shared political socialization that somehow has a long-lasting impact on their attitudes towards immigration.

The term ‘political socialization’ connotes a process of adaptation that involves the perpetuation of principles, ideals, and norms from one generation to the next. While a typical setting for this occurs in the family, the national political environment also matters. Young people are exposed to normative ideals and principles via the “political tenor” of the larger society (Levin 1961). The role of the political climate in socialization is not a new idea—yet surprisingly little effort has been made to understand the content and contours of its influence. Establishing this is not immediately apparent since “the differences between the political environments are not always dramatically large: adjacent cohorts may not have experienced sets of political events substantially different in their central political meaning” (Cutler 1976, p. 189). In our view, cohort differentiation in political behaviour does not necessarily require sharp discontinuities in the political environment, such as landmark events or regime change, which have previously drawn the attention of scholars. Without dismissing the impact of landmark events on cohorts’ attitudes, we argue that socialization can also proceed through the “fits and starts” (Sears and Valentino 1997, p. 46) as political values ebb and flow between electoral cycles.

In this article, we examine the role of the political climate during formative years as an overlooked reason as to why differences in attitudes towards immigration emerge across cohorts and persist later in adulthood. By political climate, we refer to the prevalence of certain principles, norms, and ideas in the polity and party elites at a given point in time. Existing research on the formation of attitudes towards immigration or ethnic minorities tends to focus on how social climates, such as the family (Dinas and Fouka 2018; Miklikowska 2016), peers (Aboud and Amato 2008), or school (Lancee and Sarrasin 2015; Thomsen and Olsen 2017) act as socializing agents. Our aim here is not to deny the role of these already established micro- and meso-level contexts as socializing agents. We appeal to the notion that individuals are subject to simultaneous contexts of influence during their socialization.^1^

We theorize that discontinuities in the prevailing principles of equality and tradition during a person’s formative political climate impinge on their attitudes towards immigration as adults. From this, we derive hypotheses that we test using historical political data from the Manifesto Project Dataset that we integrate with contemporary micro-data on attitudes towards immigration from the European Social Survey (2002–2020) across thirteen cohorts in thirteen European countries. To model the potential effect of the political climate during the respondents’ formative years, we conduct a hierarchical age-period-cohort analysis with synthetic age cohorts. Our research design allows us to compare attitudes to immigration between cohorts socialized during the years 1949–2018.

Our contribution to the scholarly literature is twofold. Firstly, with notable exceptions (Grasso et al. 2019; Smets and Neundorf 2014), the political climate of the larger society—that is the country as a whole—during a person’s youth has been an understudied aspect of the political socialization process. By focusing on early socialization, we contribute by theorizing how the political climate of a person’s formative years becomes an important antecedent to their attitudes towards immigration later in life. Secondly, we also make a contribution to the scholarly understanding of attitudes towards immigration. We do so by empirically demonstrating what factors contribute to the formation of immigration attitudes during a person’s youth and how these produce systematic differentiation between cohorts. While earlier studies have noted a pattern (for instance Ford (2011) in Britain), the reasons for it are not well understood and the topic is still in its infancy.

## The political socialization of cohorts

Political orientations tend to be acquired during a person’s impressionable years, a critical period of young adulthood. Individuals experience a finite period of ‘plasticity’ while they transition from adolescence to young adulthood as they engage for the first time with social and political institutions (Hanks 1981; Marsh 1971; Neundorf et al. 2013; Niemi and Sobieszek 1977; Sapiro 2004). Due to this, political socialization, the process through which an individual “acquires his political orientations, his knowledge, feelings, and evaluations of the political world” (Dawson et al. 1977, p. 33) typically occurs during this time and reflects the adaptation of a person to their wider societal context.

The age stability argument postulates that the political predispositions a person adopts in their youth are then ‘crystallized’, meaning that they are remarkably persistent as the person grows older. As a result, certain political orientations are expected to be deeply entrenched and remain more or less stable over the lifetime, and rarely subjected to within individual change (Jennings and Markus 1984; Lewis-Beck 2009; Sears 1981; Visser and Krosnick 1998).^2^ Crystallization in youth tends to be the case for symbolic political issues (e.g. attitudes to minorities or views about abortion) which tend to be impervious to life-cycle events (such as becoming a parent or retiring from the labour market). That is because symbolic issues have an affective basis, unlike non-symbolic issues (e.g. preferences for tax policy) which are more likely to have a cognitive and informational basis and therefore are more responsive to material changes over a person’s life (Henry and Sears 2009).

In recent years, a person’s attitudes towards immigration have come to be understood as a largely symbolic political issue.^3^ In this view, people’s opinions about immigration are driven by their reactions to immigrants as symbols that threaten national culture, identity, and a person’s sense of belonging (McLaren 2007; Schildkraut 2010, 2014). Given the symbolic motivation of attitudes towards immigration, it is then likely that they differ between individuals and that these differences then persist across the lifetime. In fact, previous empirical studies show that attitudes towards immigration are stable through s adulthood (Hooghe and Wilkenfeld 2008) in a similar way to other group-related attitudes such as those towards ethnic minorities (Sears and Funk 1999). In line with this, some studies demonstrate that retiring from the labour market, an important life-cycle event, does not affect a person’s attitudes towards immigration (Jeannet 2018).

Assuming that attitudes towards immigration are formed quite early in life and persist over a lifetime, we would then expect to observe a systematic pattern in political attitudes across cohorts. In other words, as attitudes are understood to be “stamped” in young adulthood, each age cohort has a different stamp due to different tempo-spatial contextual environments in which they came of age (Schuman and Corning 2012; Schuman and Rodgers 2004; Schuman and Scott 1989). Through this phenomenon, systematic differences emerge in values, beliefs, and attitudes between cohorts that persist as they grow older (Abramson and Inglehart 1992; Inglehart 2008).

## The role of the formative political climate

Our study sits within an emergent strand of literature that has emphasized a top-down and gradual approach to political socialization, distinguishing itself from other strands which have emphasized the importance of key historical events.^4^ In this view, political socialization is not only the product of landmark events which are perceptible and easily articulated by those who experienced them in their youth. Rather, political contexts can socialize through gradual ‘permeation’ or the ‘trickle-down’ of values in slow-moving processes (Grasso et al. 2019; Gray et al. 2020; Pierson 2004). Empirical evidence now shows that fairly mundane short-term factors can also have distinct long-lasting effects on political behaviour (Gomez 2021; Smets and Neundorf 2014).

We build on Grasso et al. (2019)’s notion that a diffuse political context in which a cohort came of age is a source of latent political socialization. This is in contrast to previous more narrow interpretations, which focus on exposure to specific political events or regime change affecting attitudes to immigration. For example, Abrajano and Lundgren (2014) demonstrate how landmark immigration legislation that was enacted during a cohort’s impressionable years then influenced its immigration attitudes later in life. Yet, landmark events are not a sufficient general explanation for inter-cohort variation in attitudes, as even age-cohorts who came of age in the absence of landmark political events still exhibit distinctive attitudes to immigration (Gorodzeisky and Semyonov 2018). Existing research regarding public attitudes towards immigration, albeit sparse, provides support for the plausibility of the political climate’s effect. For instance, a recent study links the mobilization of far-right political elites to the resurgence of anti-immigration attitudes in younger generations (McLaren and Paterson 2019). Another study by Wilkes and Corrigall-Brown (2011) implies that cohort differences in Canada may be due to the government’s change in discourse which highlights the beneficial aspects of immigration.

Drawing on both the empirical evidence of the contemporary political context on a person’s political behaviour (Conway 1989; Layman and Green 2006; Newman 2013) and the theoretical understanding of the political socialization of age-cohorts during the impressionable years, we argue that the political climate during a person’s formative years is an influential socializing agent in addition to their family, school, and peer group. We define political climate as an ensemble of normative principles, beliefs, ideals, and values that prevail in the political zeitgeist and therefore are reflected in the opinions of the polity and the discourse of the party elites. In our case, we focus on the formative political climate, which is the political climate during a person’s impressionable years as opposed to the contemporary political climate.

We put forward two principles that exist in the national political climate, which we deem most likely to be related to the formation of attitudes towards immigration. We use the term principle as synonymous with societal values. Scholars of values often use the word ‘principles’ to define what values are. For instance, scholars define values as goals which ‘serve as guiding principles in the life of a person or other social entity’ (Schwartz 1994, p. 21) and are the principles upon which the formation of ‘concrete attitudes toward particular objects’ occurs (Rokeach 1968, p. 550). Attitudes are distinct from values as they are ‘the evaluative sum of several beliefs with respect to a certain object’ (Fishbein and Ajzen 1975; Davidov et al. 2020, p. 584) such as immigration.^5^

There is an existing body of work that has advanced connection between values and attitudes towards immigration (Araújo et al. 2020; Davidov et al. 2020; Davidov and Meuleman 2012; Davidov et al. 2008). According to the logic of value theory put forward by Davidov and his collaborators, certain values motivate the need to ‘understand other people and show tolerance to them’ which makes individuals feel less threatened by immigration and more likely to support it. On the contrary, other values motivate the protection of ‘customs and traditions and resist violations of social expectations or norms’ which motivates the formulation of opposition to immigration as it is perceived as a greater social threat’ (Davidov et al. 2020, p. 556).

The phenomenon of immigration is related to the pursuit of two principles, but in opposing ways. The principle of maintaining tradition is challenged by the arrival of immigrants, who bring their own norms and traditions, changing their host society in the process. On the other hand, the principle of equality is bolstered by the arrival of immigrants, since this allows for the expression of understanding, acceptance, and tolerance of “others” (Davidov et al. 2008). Both of these principles are closely connected to certain human values proposed by Schwartz’s (1994) value theory which prior research has demonstrated are powerful forces in shaping attitudes towards immigration.^6^ Schwartz (1994) proposes ten different unified value concepts (universalism, benevolence, tradition, conformity, security, power, achievement, hedonism, stimulation and self-direction). Davidov et al. (2020) show that the most relevant of the two of these unified value concepts that shape attitudes to immigration are universalism and tradition. According to Schwartz (1994) tradition is “respect, commitment and acceptance of the customs and ideas that traditional culture or religion provide" whereas universalism is “understanding, appreciation, tolerance and protection for the welfare of all people and for nature” (Schwartz 1994, p. 35). While Schwartz names his value concept “universalism”, it is clear from its definition that it is tapping into the importance of equality in society.

Both the principles of equality and tradition are fundamental ideals in politics (Dahl 2006; Schaefer 2007). The phenomenon of immigration is related to the pursuit of both of these principles, but in opposing ways. The principle of maintaining tradition is challenged by the arrival of immigrants, who bring their own norms and traditions, changing their host society in the process. On the other hand, the principle of equality is heightened in a society where differences are accepted and immigrants are welcomed, allowing for the expression of understanding, acceptance, and tolerance of “others” (Davidov et al. 2008).

Through the process of political socialization, a political climate with a heightened principle of equality is expected to foster the formulation of positive attitudes towards immigration, while a political climate with a heightened principle of tradition is expected to foster the formulation of negative ones. We expect the underlying mechanism to act through the person’s normative adaptation to those principles that are hegemonic in politics at the time. We do not mean this in a simplistic sense, whereby a formative political environment turns young people into fully fledged egalitarians or traditionalists. Rather, the logic of our argument is somewhat more nuanced. According to our line of reasoning a young person who grows up in a political environment with strong prevailing principles of equality is less likely to express negative views towards immigration than if she had come of age in a political milieu where the discourse is dominated by traditional principles.

The relative importance of principles of equality and tradition in popular party discourse tends to oscillate temporally along with the thermostatic opinions of the polity (Stimson 1999). This fluctuation provides variation in the extent to which cohorts are exposed to these principles during the formative years. We expect that the variation in this exposure then explains systematic patterns in attitudes towards immigration across cohorts later in life. It is also important to clarify that we do not expect the two principles to be direct substitutes, meaning that in a political climate where the principle of equality is heightened this does not necessarily mean that the principle of traditionalism is not present as well.

Based on our theoretical framework, we derive two testable hypotheses:

### Hypothesis 1

Individuals belonging to a cohort that experienced a formative political climate where the principle of equality was heightened are significantly more likely to express support for immigration than individuals belonging to other cohorts.

### Hypothesis 2

Individuals belonging to a cohort that experienced a formative political climate where the principle of tradition was heightened are significantly less likely to express support for immigration than individuals belonging to other cohorts.

The stated hypotheses reflect the goal of this study which is concerned with the impact of the socialization environment on cohorts’ enduring attitudes. For this reason, we do not put forward hypotheses regarding other temporal dimensions of immigration attitudes such as period effects (e.g. a particular year) or the effect of a person’s age at the time of the survey as they are unrelated to a person’s formative environment. As they are formulated, these hypotheses will test the differences between cohorts that persist over time, rather than the change of attitudes within a cohort over time (for an overview of this distinction see Hamaker (2015)). We explain how we empirically isolate the cohort effect from age or period in the methods section below.

## Alternative explanations

To properly test our hypotheses, we must also exclude other alternative explanations for systematic cohort variation in attitudes towards immigration. To do so, we begin by identifying other possible factors which set younger cohorts apart from older cohorts, which might explain their systematic differences in attitudes towards immigration to then exclude them empirically. Other possible explanations could be found in the socio-economic and demographic features of the cohorts themselves. Firstly, the systematic presence of migrants and individuals with an immigrant heritage in younger cohorts compared to older cohorts generates important compositional differences with substantial attitudinal consequences. For the most part, younger cohorts in Europe have a greater proportion of first or second-generation immigrants, a social group that has been found to have more positive sentiments about immigration (Hjerm 2009).

Secondly, younger generations are simply more heavily comprised of university-educated individuals than older generations. Differences in educational attainment across generations are highly relevant for attitudes towards immigration, given the well documented positive ‘educational effect’ (Hainmueller and Hopkins 2014). Finally, the role of coming of age during economically difficult times (such as moments of high unemployment rates) also has a formative effect on attitudes towards immigration (Gorodzeisky and Semyonov 2018).

## Data and method

### Data

Our interest is in explaining differences in individual attitudes to immigration across cohorts within countries. The complexity of our design requires an accurate specification of influential factors at each level of analysis. To test our hypotheses, micro-level data that include measures of attitudes to immigration at the individual-level as well as contextual-level data for cohorts and survey years in each country are required. In order to assess the contextual socialization effect during respondents’ formative years, we collect indicators that capture historical characteristics of interest (at the time when respondents were 18 years old) in each country. It is important to point out that this operationalization assumes that each respondent was socialized in the country in which he or she now lives.^7^ Although not directly connected to our hypotheses testing, we also control for macro-level indicators at the time when surveys were conducted in each country to capture the current macro-level effects that affect all cohorts similarly.

At the individual level, the present analysis relies on biannual data from the European Social Survey (ESS) for the period 2002–2020 in thirteen European countries across 169 country-cohorts (European Social Survey 2018).^8^ The ESS survey instrument has been widely used by scholars to measure attitudes towards immigration.^9^ Using the ESS allows us to disentangle the effect of age, cohort and time period on attitudes to immigration across a number of European countries because people of the same cohort in one country are observed at different stages of their life as well as at different time periods. Moreover, using cross-sectional data we are also able to observe different formative climates during the same time period. We integrate the micro-attitudinal data from the ESS with contextual data at the cohort and period levels. These are gathered from various sources, which are further described below.

The number of countries in our sample is restricted according to several criteria. First, we include only countries that have participated in at least five rounds of the ESS to sufficiently estimate period effects which require the longest possible temporal variation. Second, only countries for which data regarding our key independent (individual, cohort and period level) variables were available are included. Finally, we included only countries that have had a democratic political regime since 1945.^10^ We are restricted by the lack of political party data for these countries but it also has the substantive advantage of ensuring that all included countries have a possible fluctuation of the prevalent political climate over the years typically associated with a multiparty system compared to dictatorial (one-party) systems. Thus, the final sample of countries includes Austria, Belgium, Switzerland, Germany,^11^ Denmark, Finland, United Kingdom, Ireland, Iceland, Italy, Netherlands, Norway and Sweden.

The sample is restricted to respondents born between 1931 and 2000 and to those who were between 18 and 85 years old in the year of the survey. These restrictions are imposed for several reasons. Firstly, we aimed to have each cohort represented in as many periods as possible.^12^ Second, we expect respondents younger than 18 years old not to have had the chance to fully socialize into the political culture and be entirely exposed to the political climate of their country. Since we are examining more complex attitudes, we would expect that political socialization and the coming of age should occur when the respondents have reached adulthood and not earlier (Bartels and Jackman 2014). Moreover, 18 years is also the age when most respondents are eligible to vote in their respective countries, presumably being more aware of the political reality compared to their younger counterparts. Finally, we necessitated a large enough number of observations for every year of age. Given the small number of individuals over the age of 85 in our sample, we eliminated respondents who are 86 years old and older, due to the uncertainty of the estimates for these cohorts.

### Measurement

Our dependent variable is a composite index that measures a person’s overall assessment of the impact of immigration on their society. Respondents were asked three questions: (1) Would you say it is generally bad or good for [country]’s economy that people come to live here from other countries? (2) Would you say that [country]’s cultural life is generally undermined or enriched by people coming to live here from other countries? and (3) Is [country] made a worse or a better place to live by people coming to live here from other countries? Answers are coded on an eleven-point scale where 0 is the most negative and 10 is the most positive reply. We created an additive index ranging from 0 to 30.^13^ The index has been widely used by other scholars studying attitudes to immigration (Gorodzeisky and Semyonov 2018; McLaren and Paterson 2019; Sides and Citrin 2007). Those respondents with missing values on any of the three items^14^ were excluded from the analysis.^15^

Apart from age, we included a set of demographic variables, such as gender, educational attainment, type of community the respondent resides in (urban versus rural), minority status, employment status, left-right political positioning and social class as controls. Educational attainment is one of the most often used predictors of anti-immigration attitudes. There is a rather strong consensus within the literature that higher educated individuals hold less anti-immigration attitudes compared to those with lower education due to two theoretically distinct causal effects (Cavaille and Marshall 2019; Drazanova 2017). On the one hand, education is thought to affect pro-immigrant attitudes of the higher educated by directly improving the knowledge (and appreciation) of foreign cultures. This effect takes place through the creation of cosmopolitan social networks by increasing exposure to different types of people, lifestyles and ideas. When individuals have greater opportunities to learn about groups different from their own, they might learn that being different is not necessarily dangerous or bad. Moreover, education is thought to develop individual cognitive competence and cognitive sophistication enabling one to better understand that the principles of democracy and equality apply to all (Prothro and Grigg 1960; McClosky and Brill 1983). On the other hand, since numerous studies confirm that higher educated individuals are usually part of a higher socio-economic class with financial security, they do not directly compete on the labour market with, for example, often low skilled ethnic minorities. Thus, higher educated individuals are less inclined to keep social distance from minorities because they do not perceive them as a threat (Bowles and Gintis 1976).

Members of the working class and those in economic distress are thought to hold more authoritarian and less tolerant values towards outgroups and minorities than those from the middle-class (Svallfors 2006) since those are directly economically threatened by them. Previous studies (Burns and Gimpel 2000; Espenshade and Hempstead 1996) have found that a perceived economic competition in the form of a pessimistic personal economic outlook leads to greater negativity towards immigrants compared to an actual one (Espenshade and Calhoun 1993). We, therefore, control for objective social class based on the Oesch class schema (Oesch 2006), employment status as well as the subjective perception of income difficulties.

Studies generally assume men should hold more anti-immigration attitudes, due to their more authoritarian personalities and conservatism (Harteveld et al. 2015) as well as their higher propensity to vote for radical right populist parties (Givens 2004). Individuals living in urban areas are often predicted to hold more positive immigration attitudes. This is theoretically based on the contact hypothesis or compositional effects (Maxwell 2019). Citizenship and minority status are included in models of attitudes to immigration based on the idea that immigrants and ethnic and racial minorities are more favourable to immigration because they can identify more strongly with other immigrants due to their own migration history (Becker 2019) or due to their similar outgroup status. Ford (2011) shows, at least for the UK, that also certain attitudes such as authoritarian-libertarian values and attitudes to ethnic diversity can potentially impact attitudes to immigration.

Following standard practice in age-period-cohort models (Reither et al. 2015), we divide the survey population into five-year country-cohorts, where individuals in the sample are nested in thirteen cohorts based on their year of birth. The cohorts’ birth years range from 1931–1935 to 1996–2000. We excluded respondents with missing data in the individual independent variables used in the regression analysis. The final sample is thus 148 690 respondents. The list of countries, country codes, and the total sample size for each country as well as for each cohort and each ESS round are available in Table A1.3 in the supplementary information.

#### Cohort-level variables

To test our expectations regarding systematic cohort differences in attitudes to immigration, we introduce a series of country-cohort independent variables into our model. Firstly, information for all independent variables in each country was gathered at the time respondents were 18 years old. Secondly, we then take the average across all years when respondents from one cohort were 18 years old to obtain a single value for each indicator of interest. For instance, for the oldest cohort (born between 1931–1935) in Belgium, any given country-cohort independent variable is calculated as the mean value of the independent variable in the years 1949, 1950, 1951, 1952 and 1953 in Belgium. This way, we are allowing the country-cohort variables to have an effect beyond one’s specific birth year and we average the country-cohort variables for the ages 18–23 in each cohort.

We hypothesize that fluctuations in principles of equality (H1) and tradition (H2) in the formative political climate explain the systematic differences in attitudes towards immigration across age-cohorts later in life. We, therefore, look at the presence of the principles of equality and tradition during times when the respondents in our sample were socialized. We rely on data from The Manifesto Project, a widely used data set that includes a coded content analysis of party manifestos since 1945.^16^ Using this data, we measure the two principles as the share of quasi-sentences calculated as a fraction of the overall number of allocated codes per manifesto (Volkens et al. 2018). The principle of equality is understood as a positive concept of social justice and the need for fair treatment of all people.^17^ On the other hand, the principle of tradition is coded as positive or favourable mentions of traditional and/or religious moral values.^18^

**Fig. 1:**
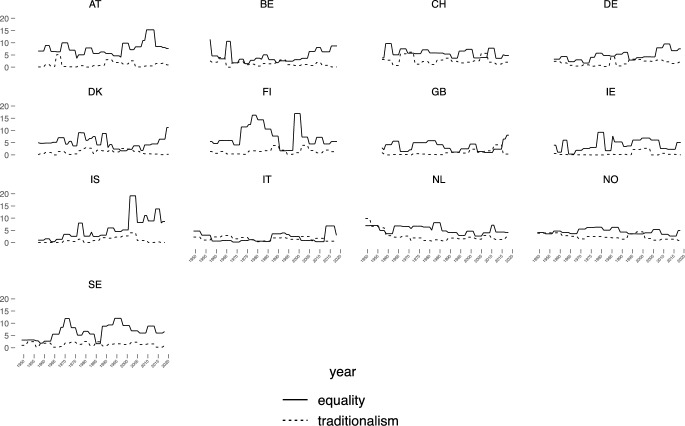
Equality and tradition in political climate over time

We do not expect the two principles to be in sharp antagonism between each other. The two principles can coexist at the same time, while, during different periods, one is more pronounced than the other. When trying to confirm our expectations empirically, as a first step we look at longterm trends in equality and tradition across countries (see Fig. 1).

Figure 1 shows clearly distinctive patterns for both of the principles in each country (an increase in one principle does not necessarily lead to a decrease in the other principle).^19^

As the data in the Manifesto Project is provided at the political-party level, we transform it into an annual measure by country. To do so, a weighted average was calculated for each country-year. We weighted by the share of votes that the party has received in the country’s last elections for two main reasons.^20^ Firstly, this type of weighting accounts for the popularity of the party and how likely it is that said party’s manifesto and general preferences will receive attention in the country (for example through the media). It is easy to imagine that in a country with few niche parties promoting the ideal of equality, but also with one major party promoting the value of tradition, the relative electoral support for the parties would matter for the general political climate and that the general political climate would probably be more traditionalist than egalitarian. Secondly, while taking into consideration the relative electoral importance of the party, we account for both basic ways of how politics operates; the fact that political parties influence the fundamental principles which emerge in the political tenor (supply-side), but also the fact that these principles may be more or less upheld by the citizens (demand-side). More generally, cohorts’ attitudes could be affecting both the demand and supply component of the tradition and equality measure—vote shares expressed for parties could be reflecting cohort’s attitudes while at the same time party manifestos respond to the attitudes of the electorate. We try to overcome this endogeneity problem by concentrating on the principles of equality and tradition in the political climate when individuals in each cohort were 18–23 years old. By doing so, we assume that at this age most of the individuals have not yet had the chance to vote or have only recently been first time voters. Since parties cannot reflect these cohorts’ preferences, given that those preferences are yet to be proclaimed in (future) elections or could not be accounted for by parties in such a short time, we are avoiding the problem of reversed causality and studying the effect of the political climate on individuals, and not party responses to the overall political preferences of these cohorts.

Based on all of the above, we calculate the weighted mean of equality/tradition principles in the formative political climate when cohorts were between the ages of 18 and 23. For instance, for the cohort born between 1931 and 1935, we calculate the weighted mean of the emphasis on equality and/or tradition in political manifestos in each country for the years 1949–1953. The time trends in equality and tradition across cohorts in the countries included can be found in the supporting information in Figure A2.1 and Figure A2.2 respectively.

Apart from coming of age during distinct political climates, there might be other cohort-level factors accounting for the variation in cohorts’ immigration attitudes. Overall, educational attainment has increased in the last decades in all European countries, (Lutz et al. 2019) while higher education has also been found to have positive effects on attitudes to immigration (Cavaille and Marshall 2019; Drazanova 2017; Lancee and Sarrasin 2015). Thus, one would expect higher levels of education amongst younger cohorts to play a role in inter-cohort differences in attitudes to immigration.^21^ In order to test whether cohort differences in attitudes to immigration are due to demographic differences regarding their level of education, we calculated the percentage of university-educated individuals within each cohort.^22^

We expect growing up with different degrees of ethnic diversity to play an important role in intra-cohort differences in immigration attitudes (Allport 1954; Pettigrew et al. 2011). To measure the extent of cohorts’ exposure to immigration diversity during their formative years, we include data on countries’ net migration, provided in five-years intervals.^23^ We, therefore, assigned each cohort a net migration value in their respective country for the period when individuals in each cohort were between 18 and 23 years old.^24^

Cohorts entering the labour market when unemployment rates were high were found to be more likely to hold negative attitudes toward immigrants (Gorodzeisky and Semyonov 2018). Therefore, we also control for countries’ unemployment rate at the time each birth-cohort was 18 years old as a proxy for entering the country’s labour market. We draw on data from the OECD’s Annual Labour Force Statistics, which provides the rate of unemployment as the percentage of a country’s civilian labour force since 1956 (Organisation for Economic Co-operation and Development 2019). Again, we calculated the unemployment rate for each cohort as the mean value of the unemployment rate in the years when each individual within a cohort was 18 years old.^25^

#### Period-level variables

Certain periods might exert a shift in attitudes for all individuals in society, regardless of age or birth cohort. Therefore, in order to properly identify cohort effects and disentangle them from eventual period effects, we also need to control for period effects in our models. As the effect of time-varying processes might be different in individual countries, we control for period effects with a series of country-period independent variables.

As with the country-cohort level variables, we include two country-period variables measuring the relative dominance of equality and tradition in the contemporary political climate. The relative dominance of the principles of equality and tradition is calculated in a similar way to the case of country-cohorts, but corresponds to the year in which the survey took place in each country. To measure diversity, we apply the estimates of net migration for each country in the corresponding year of the survey, retrieved from Eurostat. Data regarding the harmonized unemployment rate were taken from OECD’s Labor Market Statistics^26^ and reflect the total percentage of unemployed labour force in each country. Variable coding and descriptive statistics of all variables are available in Table A1.1 and Table A1.2 in the supplementary information.

#### Methods

Research on cohort effects needs to address the potentially confounding influences of age and period effects when estimating models. In the literature, this issue is recognized as the age-period-cohort “identification problem” and is well known in studies of this type (McKenzie 2006; Yang et al. 2008). The identification problem emerges because age, period and cohort effects are linear functions of one another. As soon as we know two values, we simultaneously know the third, since age = period (year of survey)—birth year.

Our empirical strategy overcomes the identification problem by conducting a hierarchical age-period-cohort regression analysis (HAPC), which is well suited for repeated cross-sectional survey designs.^27^ HAPC analysis distinguishes between the three temporal phenomena of age, period (year of survey) and birth cohort (year of birth) effects using micro-data (Yang and Land 2013). It constructs synthetic cohorts based on age groups to compensate for the absence of longitudinal data, while individuals are cross-classified^28^, nested in both country-period and country-cohort.^29^

We group cohorts into equal five-year intervals based on birth as this is the standard in APC analysis (see Mason and Fienberg 1985). The reason for constructing five-year cohorts is so that the model is no longer perfectly collinear—when knowing the cohort and period, one cannot determine the exact age of the respondent, but only a range of possible ages. Moreover, constructing cohorts that include several birth years is consistent with our theoretical expectations that there are no sharp differences between individuals born in one year compared to another, “but that distinctions are caused by relatively small changes over time such that meaningful divisions are only observed between those whose formative years are temporally distant from one another” (Down and Wilson 2013, p. 438). In our case, this means that individual respondents can potentially belong to different combinations of country-cohorts and country-periods.

Taking into consideration all of the above, we apply a hierarchical three level age-period-cohort model, where individuals are nested simultaneously within two second-level variables (country-cohort and country-period) as well as nested within countries, since possible clustering at the country level might still occur. We also include random effects for cohorts and periods within countries in our model.

The level-1 model is:

1 $$\begin{aligned} Y_{ijkc} = \beta _{0jkc} + \beta _1X_{ijkc} + e_{ijkc} \end{aligned}$$

where, within each country-cohort *j,* country-period k and country *c*, respondents’ attitudes to immigration (Y) are a function of their individual characteristics (vector X). $\beta _{0jkc}$ is the mean of attitudes to immigration of individuals in country-cohort *j*, country-period *k*, and country *c,* $\beta _{1}$ is the level-1 fixed effects and $e_{ijkc}$ is the random individual variation.

The level-2 model is:

2 $$\begin{aligned} \beta _{0jkc} = \gamma _{0jkc} + C_{0jc}Z_{jc} + K_{0kc}T_{kc}+ \mu _0{jc} + \nu _{0kc} \end{aligned}$$

where Z is a vector of country-cohort characteristics and T is a vector of country-period characteristics, $\mu _0{jc}$ is the residual random effect of country-cohort *j*, $\nu _{0kc}$ is the residual random effect of country-period *k*.

The level-3 model is:

3 $$\begin{aligned} \gamma _0 = x_{0c} + \omega _{0c} \end{aligned}$$

where $\omega _{0c}$ is the residual random effect of country *c.* In all three models (1), (2) and (3) $\mu _{0j}$, $\nu _{0k}$ and $\omega _{oc}$ are assumed normally distributed with mean 0 and variance $\tau _\mu$, $\tau _\nu$ and $\tau _\omega$respectively.

A cross-classified equation for our specific model explaining attitudes to immigration can be found below. For simplicity, we show the model expressed using classification notations instead of standard hierarchical notations.

$$\begin{aligned} \begin{aligned}&\text {Attitudes to immigration}_{i} = \beta _{0} + \beta _{1}\text {age}_{i} + \beta _{2} \text {lower secondary education}_{i} \\&\quad + \beta _{3}\text {upper secondary education}_{i} + \beta _{4}\text {some university without degree}_{i} + \beta _{5}\text {university}_{i} \\&\quad + \beta _{6}\text {female}_{i} + \beta _{7}\text {urban}_{i} + \beta _{8}\text {income difficulties}_{i} + \beta _{9}\text {minority member}_{i} \\&\quad + \beta _{10}\text {unemployed}_{i} + \beta _{11}\text {left-right scale}_{i} + \beta _{12}\text {lower-grade service class}_{i} \\&\quad + \beta _{13}\text {small business owner}_{i} + \beta _{14}\text {skilled worker}_{i} + \beta _{15}\text {unskilled worker}_{i} \\&\quad + \beta _{16}\text {political climate of equality}_{country-cohorti} \\&\quad + \beta _{17}\text {political climate of tradition}_{country-cohorti} \\&\quad + \beta _{18}\text {university educated}_{country-cohorti} + \beta _{19}\text {net migration}_{country-cohorti}\\&\quad + \beta _{20}\text {unemployment rate}_{country-cohorti} + \beta _{21}\text {university educated}_{country-cohorti} \\&\quad + \beta _{22}\text {political climate of equality}_{country-periodi}\\&\quad + \beta _{23}\text {political climate of tradition}_{country-periodi} + \beta _{24}\text {net migration}_{country-periodi} \\&\quad + \beta _{25}\text {university educated}_{country-periodi} + \beta _{26}\text {unemployment rate}_{country-periodi} \\&\quad + \beta _{27}\text {country2}_{0c} + \cdots + \beta _{39}\text {country13}_{0c} + \mu _{country-cohorti} + \nu _{country-periodi} + e_{i} \end{aligned} \end{aligned}$$

## Results

We begin by estimating a so-called null hierarchical three-level cross-classified model (Model 0 in Table 1). This model provides information on the variance components of immigration attitudes at each level of analysis (Level 1—individual, Level 2—country-cohort and country-period, Level 3—country). It includes only an intercept, country-cohort random effects, country-period random effects, country random effects and an individual level residual error term.

**Fig. 2:**
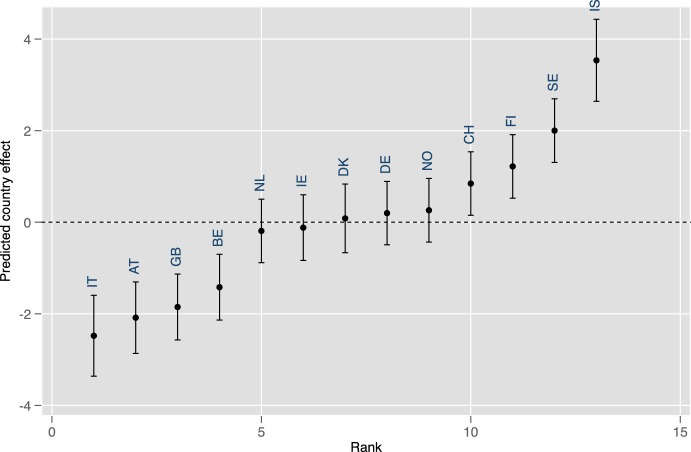
Caterpillar plot of country effects together with 95% confidence intervals

Figure 2 shows the caterpillar plot of country random effects with their associated 95% confidence intervals from the null model. Countries are shown in rank order according to their predicted effects. The horizontal zero line represents the average country in the data. As can be seen from the figure, Italy, Austria, the United Kingdom and Belgium are significantly below country average regarding their positive attitudes to immigration (averaged across cohorts and periods), while Switzerland, Finland, Sweden and Iceland hold, at the country level, significantly above-average attitudes to immigration. Other countries (the Netherlands, Ireland, Denmark, Norway and Germany) do not differ significantly from the average country.

**Fig. 3:**
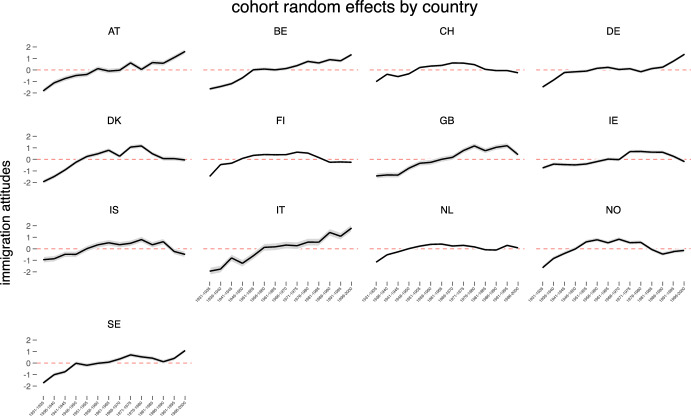
Cohort random effect estimates from the unconditional hierarchical three-level cross-classified model

**Fig. 4:**
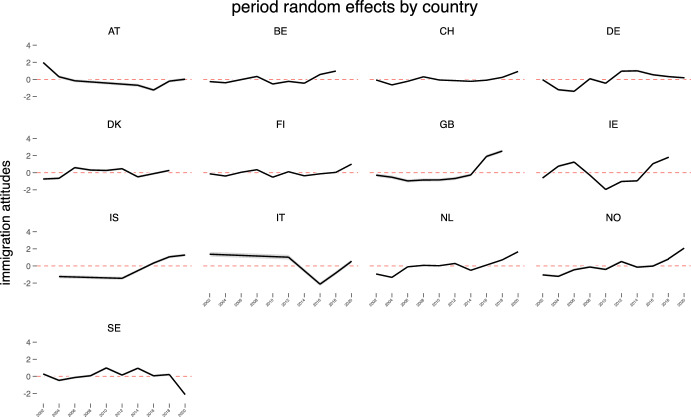
Period random effect estimates from the unconditional hierarchical three-level cross-classified model

Figure 3 and Figure 4 display the Best Linear Unbiased Predictions (BLUPs) of the country-cohort and country-period random effects from the unconditional model by country with mean equal to zero. As can be seen from Figure 3, the relationship between cohorts and immigration attitudes in many countries is not linear. While in each country the oldest generation is the most negative about immigration younger cohorts in certain countries (for example Switzerland, Norway, Finland) have more negative levels of immigration attitudes than the immediately preceding generations. These visual illustrations confirm that cross-cohort variations are rather important for understanding changes in attitudes toward immigration. Period random effects presented in Figure 4 reveal that in many countries (particularly Ireland, Germany and the United Kingdom) there are statistically significant temporal changes regarding attitudes to immigration. While in the United Kingdom and Ireland the level of attitudes to immigration became positive during the last period, in Germany pro-immigration attitudes slightly declined at the time of the latest survey (2020) compared to the previous ones (2014).

In Model 1 in Table 1 we add individual-level control variables to the null model and the one of the two variables of interest at the country-cohort level, while also controlling for the political climates of equality and tradition, unemployment and net migration at the country-period level. We present the coefficients together with the associated standard errors for the fixed part of the models as well as random coefficients for country-cohorts, country-periods and countries. Consistent with most previous studies, in general, the young are significantly more supportive of immigration than the old.^30^ Looking at the effects of other covariates, having any type of higher education compared to primary school, being male, being a member of a minority group and residing in an urban area are all significantly positively associated with immigration attitudes. On the other hand, having income difficulties, being unemployed, being part of any other social class than higher-grade service class and self-placing towards the right on a political scale are significantly negatively associated with immigration attitudes.

**Table 1:** Results of a hierarchical multilevel cross-classified model explaining cohort-differences in attitudes to immigration across 13 countries

	Model 0		Model 1		Model 2		Model 3	
	Coeff.	S.E.	Coeff.	S.E.	Coeff.	S.E.	Coeff.	S.E.
Intercept	16.90***	(0.418)	19.72***	(0.814)	19.97***	(0.813)	20.34***	(0.827)
Individual level								
Age			− 0.009***	(0.001)	− 0.010***	(0.002)	− 0.017***	(0.002)
Lower secondary education			0.691***	(0.071)	0.695***	(0.071)	0.703***	(0.071)
Upper secondary education			1.558***	(0.068)	1.562***	(0.068)	1.572***	(0.068)
University without degree			2.363***	(0.080)	2.368***	(0.034)	2.382***	(0.080)
University degree			3.604***	(0.074)	3.609***	(0.074)	3.626***	(0.074)
Female			− 0.065*	(0.030)	− 0.065*	(0.030)	− 0.064*	(0.030)
Urban residence			0.690***	(0.034)	0.690***	(0.034)	0.690***	(0.034)
Income difficulties			− 1.113***	(0.048)	− 1.112***	(0.048)	− 1.107***	(0.048)
Minority member			1.410***	(0.041)	1.410***	(0.041)	1.412***	(0.041)
Unemployment			− 0.257***	(0.068)	− 0.257***	(0.068)	− 0.257***	(0.068)
Left-right scale			− 0.483***	(0.007)	− 0.483***	(0.007)	− 0.483***	(0.007)
Lower-grade service class			− 0.730***	(0.049)	− 0.731***	(0.049)	− 0.731***	(0.049)
Small business owners			− 1.067***	(0.059)	− 1.067***	(0.059)	− 1.066***	(0.059)
Skilled workers			− 1.746***	(0.051)	− 1.745***	(0.051)	− 1.748***	(0.051)
Unskilled workers			− 1.819***	(0.059)	− 1.818***	(0.059)	− 1.821***	(0.059)
Country-cohort level								
Political climate of equality			0.028*	(0.014)			0.034**	(0.013)
Political climate of tradition					− 0.049	(0.029)	− 0.036	(0.027)
% of university educated							− 0.006	(0.004)
Net migration							− 0.035**	(0.012)
Unemployment rate							− 0.035**	(0.013)
Country-period level								
Political climate of equality			0.102*	(0.046)	0.103*	(0.046)	0.107*	(0.046)
Political climate of tradition			− 0.087*	(0.085)	− 0.086	(0.085)	− 0.096	(0.085)
Net migration			-1.03e−07	(1.53e−06)	-9.95e−0	(1.53e−06)	1.60e−07	(1.53e−06)
Unemployment rate			− 0.198***	(0.047)	− 0.200***	(0.047)	− 0.189***	(0.047)
% of university educated			− 0.014	(0.013)	− 0.014	(0.013)	− 0.012	(0.013)
Random effects								
Country	2.117	(0.913)	1.904	(0.800)	1.994	(0.816)	1.904	(0.801)
Cohort (in country)	0.480	(0.062)	0.099	(0.018)	0.098	(0.018)	0.802	(0.015)
Period (in country)	0.958	(0.148)	0.452	(0.072)	0.450	(0.072)	0.453	(0.072)
Individual	32.974	(0.133)	28.526	(0.115)	28.526	(0.115)	28.525	(0.115)

Entries are unstandardized coefficients and standard errors. ***p < 0.001, **p < 0.01, *p < 0.05 Level 1 N: 123 049 Level 2 Country-cohort N: 169; Level 2 Countryperiod N: 112; Level 3 Country-level N: 13

Recall that we hypothesized that individuals who belong to an age-cohort that experienced their formative years in a political climate dominated by the value of equality are significantly more likely to express support for immigration (H1), while individuals who experienced their formative years in a climate dominated by traditionalist values are significantly less likely to express support for immigration (H2). The significantly positive effect of equality at the country-cohort level implies that cohorts that came of age in times when the political climate in their country emphasized the value of equality are more likely to hold positive attitudes towards immigration.

Model 2 in Table 1 includes a measure of the principle of tradition in the political climate at the country-cohort level, while also controlling for individual as well as period-level factors. The negative coefficient of tradition at the country-cohort level shows that those cohorts coming of age in a political climate emphasizing traditional values are less likely to hold positive attitudes to immigration, but the effect is non-significant at conventional levels. Thefore, our hypothesis H2 is not fully supported.

Finally, Model 3 includes all independent variables at the individual level (age, gender, having a higher education than completed primary school, being a member of a minority group, residing in an urban area, having income difficulties, being unemployed, social class and left-right self-positioning on a political scale), country-cohort level (political climate of equality at the time cohorts were 18 years old, political climate of tradition at the time cohorts were 18 years old, percentage of university educated within the cohort, net migration at the time cohorts were 18 years old, and unemployment at the time cohorts were 18 years old) and country-period level (political climate of equality, political climate of tradition, net migration, unemployment rate and percentage of university educated within the country-period).

The effect of one of our two main independent variables of interest, equality at the country-cohort level, remains significant even after controlling for all other factors at different levels. The results support our argument that growing up in the political climate of equality may have a long-lasting effect on (future) political attitudes of entire generations. Those respondents who were socialized into a political climate that emphasized equality are significantly more likely to hold positive immigration attitudes compared to those who came of age in different political climates. Moreover, as the median age in the sample is 48 years old, this effect appears to be long-lasting. On the other hand, the political climate of tradition, although in the hypothesized direction, does not exert a statistically significant effect.

At the country-period level, the political climates of equality also significantly positively influence immigration attitudes, while the political climate of tradition influences it negatively (although the coefficient reaches statistical significance only in Model 1). As for other country-period control variables, unemployment has significant negative effects. Other variables do not reach the conventional level of statistical significance.

We acknowledge that both coefficients of equality and tradition in the formative political climate remain rather small but are substantively important. The inclusion of individual attributes as controls at the cohort level in Model 3 explains a greater amount of intra-cohort variation than introducing coefficients for equality and tradition in Models 1 and 2. Yet Model 3 shows that an increase in one percentage point of equality from the mean formative political climate leads to a 0.034 increase in attitudes to immigration on a scale of 0 to 30. In contrast, one percentage point increase of traditionalism from the mean formative political climate leads to a 0.036 decrease in attitudes to immigration. From a substantive view, this may represent a small effect on an individual when considered the dependent variable is measured on a scale from 0 to 30. Yet if these changes, on average, can be expected to influence an entire cohort, the cumulative importance of this effect size can be seen as more substantial and important at the macro-societal level.

The sizes of coefficients for climates of equality at the country-cohort (0.034), are notably larger than the sizes of the coefficient for country-period (0.107). Yet it is important to keep in mind these small coefficient sizes in changes in *y*, are related to relatively small changes in *x* (only a 1 percentage point increase in the political climate). It is important also to consider that while period-country political climate may have a larger association with people’s opinions about immigration, it is temporarily fleeing and is not expected to have a long last impact. On the other hand, the coefficient for the formative political climate is shown here to be small but its significance implies tenacious importance that endures later in a person’s life.

## Discussion and conclusion

Drawing on political socialization theory, we posit that a person’s formative political climate—or, in other words, the political zeitgeist during their youth—explains their attitudes towards immigration later on in life. Specifically, we hypothesize that exposure to varying levels of certain political principles in the political climate, namely equality and tradition, during a person’s youth have opposing effects on his or her attitudes to immigration in adulthood. We test our hypotheses using micro-attitudinal data that we integrated with historical political data to study over 100,000 individuals, belonging to thirteen different cohorts from thirteen European countries.

The results of the hierarchical age-period-cohort analysis presented here indicate that cohorts formulate distinct patterns of attitudes towards immigration due to a collective process of political socialization they underwent during their youth. We find empirical support for the hypothesis that exposure to a political climate fostering the principle of equality during the formative years affects a person’s attitudes towards immigration even later in life. When a person comes of age in a political climate where the principle of equality is widespread, it positively influences the attitudes towards immigration he or she has later in life. These findings are confirmed by a series of additional analyses and robustness checks, which are documented in the supporting information. We do not find evidence that coming of age in a political climate that is more steeped in the principle of tradition affects attitudes towards immigration later on in life.

Our study holds important implications regarding the sources from which a person’s attitudes towards immigration originate. Traditional analysis generally investigates the effect of contemporary politics on attitudes. In contrast, our study deviates from this to reveal the importance of yesterday’s politics on today’s attitudes. The findings indicate that contextual exposure to the principle of equality is central to the formulation of immigration attitudes, regardless of whether or not the person holds these ideals themselves. Since cohorts occupy the same temporal-spatial political context during their coming of age, their attitudes towards immigration as adults reflect this shared political socialization.

The ideals propagated by political elites and their relative popularity among the polity typically oscillate. Our findings imply that even these subtle and cyclical shifts have a formative power during the process of the political socialization of youth. We contribute to the theoretical understanding of political socialization, as we believe that the general logic of our argument should apply to other symbolic attitudes besides immigration. Importantly, our results demonstrate that cohort differentiation in political behaviour does not require radical shocks such as landmark events or regime change, albeit their effects are more conducive to an empirical identification. This implies that principles that are common in a particular political climate have an implicit normative function for those who are socialized amongst them, affecting their political behaviour later in life.

Naturally, our results are subject to some limitations. Our analysis cannot fully address what makes the principle of equality ebb and flow in the first place. We cannot entirely rule out the possibility that the principle is tied to underlying cyclical changes in the popularity of liberal and conservative ideologies. Typically, socially liberal parties tend to emphasize equality. Therefore, the importance of these ideals in the political climate is possibly correlated to the political ideology of the party that holds power. To address this and validate our findings, we conduct a falsification test and we test our same model but change the dependent variable from attitudes to immigration to attitudes about redistribution. The logic would be that, if our results were driven by underlying political ideological values, we would find similar results if we ran the model with attitudes towards redistribution since it is typically quite aligned with political value orientations. Our results ‘pass’ the falsification test, in the sense that they do not hold for attitudes towards redistribution (please see Table 3.1 in the supporting information). The analysis shows that cohort-level equality does not significantly impact these attitudes (but cohort-level traditionalism significantly negatively impacts them). Thus, the effect on attitudes to income redistribution of our main variables of interest works differently than on attitudes to immigration. We take this to be further evidence that our findings are not an artefact.

Furthermore, the drawbacks to using cross-sectional surveys which span a period of 18 years (2002–2020) mean that we have not been able to follow how attitudes towards immigration maturate across the longer course of a single person’s life or in the general public over many decades. Likewise, we are fully aware that, being a comparative study, our findings pertain to the average found across the European countries in our sample and, as such, may not apply to a specific country. We recognize that each country has a particular composition of specific nationalities or ethnic groups which can further shape attitudes towards immigration in that specific country (e.g. Ford 2011).

Finally, our results call on future research to address questions about the way in which the principles of the political tenor are diffused to young people. In our study, the data at hand does not allow us to investigate if principles of the contemporary political tenor are transmitted to young people via their parents. It would be useful for future research to examine, for instance, whether young people are exposed to these principles through the mass media or through their social environments such as peers or family. These channels may not be mutually exclusive and a fuller picture would emerge if research might explore how the principles of the political tenor are then filtered by other socializing agents as part of a simultaneous process. Moreover, future research should also corroborate and further investigate these findings in the context of single-country study designs which could allow for a more in-depth exploration of a particular national political climate.

Political socialization is about the perpetuation of ideals, norms, and principles from one generation to the next. It is, therefore, worth noting that based on our findings, we can speculate about public attitudes towards immigration in future generations. Young people are undergoing socialization in the current political environment, rendering the ideals, norms, and principles that predominate in the tenor of politics today highly relevant for tomorrow. For attitudes towards immigration to become more positive the principle of equality needs to be widespread. Looking at the current political climate situation in Europe, the future is rather foreboding, as the continued rise of the radical right-wing generates exposure to ideals and values which are antithetical to the formulation of pro-immigration views during a person’s formative years.

## Supplementary Information

Below is the link to the electronic supplementary material.

Supplementary file1 (PDF 550 kb)

## Footnotes

1 For an overview see Hatemi and McDermott (2016).

2 Still, the persistence of a person’s pre-adult attitudes through their lifetime has been debated by scholars (Alwin et al. 1991; Sears and Funk 1999).

3 The notion that attitudes to immigration are due to labour market competition or other ego-tropic cost-benefit calculations has been highly critiqued (see Hainmueller and Hopkins (2014)) although recent evidence has shown that economic conditions can matter if a person perceives their social group to be deprived of desirable resources (Meuleman et al. 2020)

4 Some studies have shown that landmark political events, such as the Watergate political scandal or the Vietnam War, experienced during youth leave their mark on a cohort’s political behaviour (Dinas 2013; Erikson and Stoker 2011; Schuman and Corning 2012).

5 Henceforth, we use the words principle, value, and ideal interchangeably.

6 Inglehart also proposes another prominent value theory (Inglehart 1977, 2008). We choose not to draw on this value theory since it is mostly oriented towards explaining postmodernism. Moreover, Inglehart’s cultural value theory has a more tenuous and less demonstrated link with attitudes to immigration compared to Schwartz’s human values theory (see Datler et al. (2013) for an overview and comparison of these two value schemes).

7 We take this into consideration in our models by controlling for individuals who are not citizens of the country in which they were surveyed and/or first or second-generation migrants and/or an ethnic minority, to minimize the possibility that they were not socialized there or that their answers might be different due to their migrant status. Moreover, we conduct several robustness checks in this regard. We estimate all the models for only respondents who have been born in the country and/or came to the country before they were 18 years old, obtaining substantially the same results. Additionally, we also estimate the main models only for respondents who have been born in the country as well as both of their parents have been born in the country and do not classify themselves as an ethnic minority, once again obtaining substantially the same results

8 We acknowledge that some might deem this a relatively short time span (18 years that include 10 observable periods) for the study of age, period, cohort effects.

9 See Hainmueller and Hopkins (2014).

10 Applying these criteria excludes countries in Western Europe such as Spain and Portugal as well as Central and Eastern European countries.

11 Due to historical disparities which likely influence differences between cohorts in the two parts of Germany, we divided Germany into East Germany and West Germany. Only respondents from West Germany have been analyzed. However, as an anonymous reviewer pointed out, it is worth mentioning that since the fall of the Berlin Wall until 2006, the net loss due to (mainly internal) migration amounted to 1.74 million people—almost one-tenth of the former GDR’s population (Glorius 2010). On the other hand, it should be underlined that between 2001 and 2006, three million departures to West Germany were paralleled by about two million moves in the opposite direction and a considerable part of this counterflow consisted of returning East German migrants (Glorius 2010)

12 Nevertheless, the two youngest cohorts are not present in every period (those born between the years 1996–2000 are not observed in the survey years 2002, 2004, 2006, 2008, 2010 and 2012 and those born between the years 1991–1995 are not observed in the survey years 2002, 2004, 2006). The oldest cohort (those born between 1931–1935) is also not present in the latest survey of 2020.

13 The McDonald’s omega for the three items is 0.85 as well as Cronbach’s alpha, thus confirming that these items measure a similar underlying concept.

14 Overall, 4.19 percent of respondents (8 104) were excluded from the analysis because of lack of information on any of the three items forming the dependent variable.

15 As a robustness check, we also re-estimated the models including respondents that answered at least two of the three items comprising our dependent variable. The results obtained from this analysis were nearly identical to those presented here.

16 The Comparative Manifesto dataset is a widely used data set in political science and its reliability has been heavily scrutinized (see Gemenis (2017)). While there are also other surveys measuring party positions, such as the Chapel Hill Expert Survey, these do not provide longitudinal data further enough to allow us to measure parties’ stances since 1945.

17 “This includes references to topics such as special protection for underprivileged social groups, removal of class barriers, need for fair distribution of resources and the end of racial or sexual discrimination” (Volkens et al. 2018, p. 17).

18 “This includes references to topics such as prohibition, censorship and suppression of immorality and unseemly behaviour, maintenance and stability of the traditional family as a value, and support for the role of religious institutions in state and society.” (Volkens et al. 2018, p. 19).

19 Apart from this visual inspection, we also use Pearson’s correlation coefficient as a way to examine these principles’ association. In none of the countries did we find a (neither positive nor negative) strong correlation between the two principles (results available upon request). These findings confirm our expectation that the two principles of equality and traditionalism are not necessarily perceived by parties (and citizens) as a trade-off between each other and thus manifest distinct patterns.

20 In the case of mixed electoral systems with a proportional and majoritarian component, we use the vote share in the proportional component. In the case of an electoral coalition where programs for all members of the coalition and the coalition were coded, we set the vote share to zero for the coalition program so that the sum of the share is not higher than 100 percent.

21 We would like to note that the two youngest cohorts (those born between the years 1990–1995 and 1996–2000) have a lower percentage of university-educated individuals compared to older cohorts because in many instances (for example in the 2002 ESS survey year), they were too young to have completed a university degree.

22 We opted to calculate the percentage of university-educated individuals within each cohort instead of the cohort’s mean years of education. Years of education do not provide enough information about the respondents’ educational level, since they may not be comparable across countries, especially if the countries’ respective educational systems are too different.

23 Data is made available by the United Nations Department of Economic and Social Affairs’ Population Division (2020) since 1950.

24 For example, for the oldest cohort born between 1931–1935 we assigned the estimated net migration for the years 1950–1955, while we attributed the net migration estimate for the years 1955–1960 to the cohort born between 1936–1940.

25 For the oldest cohort (1931–1935) we used only the value of the unemployment rate in 1956, because of a lack of comparable data in previous years.

26 Data for Switzerland in the years 2002–2008 come from Harmonized Unemployment Rate: All Persons for Switzerland fourth quarter.

27 We are aware that, as has been the case with all of the widely used APC methods, HAPC modelling has been subjected to debate and critique (most famously, by Bell and Jones 2018). The main criticism that Bell and Jones (2018) raise regarding HAPC models is that they can be biased and misleading when the dependent variable follows period or cohort near-linear trends (i.e. a continuous upward/downward trend). Therefore, to confirm the suitability of HAPC modelling in our study, we take steps to rule out that such linear trends might exist in our sample. A visual inspection of Figure 3 and Figure 4 demonstrates that neither cohorts nor periods in our sample follow a consistent linear trend, both across countries and within each country. Moreover, we also replicate the analysis using an alternative approach. One of the alternative approaches to APC models used by researchers is the ‘drop-one’ variable, which deals with the age-period-cohort collinearity problem by dropping one of the three. We test this by dropping age from the model and measuring age effects indirectly by using underlying indicators of social aging. We follow Schmidt (2021) and add the variable whether a respondent is currently, or has ever been, married or lived in a civil partnership (coded as “yes” (0), “no” (1)), as a proxy for the effect of aging. Please look at Table 5.1 in the supplementary material. The results are consistent.

28 In cross-classified data, lower level units do not belong to one and only one higher level unit. Rather, lower-level units belong to pairs or combinations of higher-level units formed by crossing two or more higher-level classifications with one another.

29 Following Yang (2008), we assume that while age is related to biological processes of aging, cohort and period effects rather reflect the influences of external forces and as such should be considered as macro-level variables.

30 We also estimated the same models including age squared. However, as in none of the models the variable has been significant and its substantial effect has been very small, we decided not to include it in our main models, as it is not standard to include age squared in HAPC models.
