# Supplementary material for: Blame it on my youth: the origins of attitudes towards immigration
Source: Acta Polit. 2023 Oct 28;59(4):866–95. doi: 10.1057/s41269-023-00314-6 (PMC11412906; doi:10.1057/s41269-023-00314-6)
Supplement: Supplementary file 1 — Supplementary file1 (PDF 550 kb) [file 41269_2023_314_MOESM1_ESM.pdf]

Blame It On My Youth: The Origins of Attitudes Towards Immigration

Supporting Information

Table of contents

Table of contents 2
Appendix 1. Coding and descriptive statistics of variables 3
Appendix 2. Historical evolution of the presence of the principles of equality and tradition across cohorts in the countries included 6
Appendix 3. Robustness of the results when using a different dependent variable (income redistribution) 8
Appendix 4. Robustness of the results when inserting measures of libertarian-authoritarian values 9
Appendix 5. Robustness of the results with a different specification model 10

Appendix 1. Coding and descriptive statistics of variables

Table A1.1 Variable coding

Variable	Coding
Dependent variable	
Attitudes to immigration	Additive (continuous) index consisting of answers to three questions:<br>(1) Would you say it is generally bad or good for [country]'s economy that people come to live here from other countries?<br>(2) Would you say that [country]'s cultural life is generally undermined or enriched by people coming to live here from other countries?<br>(3) Is [country] made a worse or a better place to live by people coming to live here from other countries?
Individual-level	
Age	Continuous measure of the age of respondent
Less than lower secondary	*Reference category*
Lower secondary education	1= Lower secondary education 0 = no lower secondary education
Upper secondary education	1= Upper secondary education 0 = no upper secondary education
University without degree	1= University without degree, 0 = No university without degree
University degree	1= University degree, 0 = No university degree
Female	1 = Female, 0 = Male
Urban residence	1 = urban residence (big city or suburbs or outskirts of big city), 0 = rural residence
Income difficulties	1 = respondent finds it a) difficult or b) very difficult to live on current household´s income, 0 = respondent is a) coping on present income or b) living comfortably on present income
Minority member	1 = respondent first or second-generation migrant, and/or not a citizen of the country and/or ethnic minority, 0 = otherwise
Unemployment	1 = Respondent unemployed, 0 = respondent not unemployed
Left-right scale	Continuous measure where 0 = left … 10 = right
Higher-grade service class	*Reference category*
Lower-grade service class	1= Lower-grade service class, 0 = not lower-grade service class
Small business owners	1 = Small business owner, 0 = not small business owner
Skilled workers	1 = Skilled worker, 0 = not skilled worker
Unskilled workers	1 = unskilled worker, 0 = not unskilled worker

Table A1.1 Variable coding – *Continued from previous page*

Variable	Coding
Country-Cohort level	
Political climate of equality	Continuous measure of emphasis on equality in parties´ political manifestos in each country annually weighted by the percentage of seats in Parliament by respective party calculated as a mean value for each year when each respondent forming one cohort were 18 years old in their respective country, retrieved from the Manifesto Project
Political climate of tradition	Continuous measure of emphasis on tradition in parties´ political manifestos in each country annually weighted by the percentage of seats in Parliament by respective party calculated as a mean value for each year when each respondent forming one cohort were 18 years old in their respective country, retrieved from the Manifesto Project
% of university educated	Continuous measure of the percentage of university educated individuals within each country-cohort calculated directly from the ESS
Net migration	Continuous measure of net migration in the 5-years respondents were between 18 and 23 years old in their respective countries, retrieved from the United Nations Department of Economic and Social Affairs´ Population Division
Unemployment rate	Continuous measure of the percentage of country's civilian labour force averaged across all country-cohorts when respondents were 18 years old, retrieved from the OECD´s Annual Labour Force Statistics
Country-Period level	
Political climate of equality	Continuous measure of emphasis on equality in parties´ political manifestos in each country in survey year weighted by the percentage of seats in Parliament by respective party, retrieved from the Manifesto Project
Political climate of tradition	Continuous measure of emphasis on tradition in parties´ political manifestos in each country in survey year weighted by the percentage of seats in Parliament by respective party, retrieved from the Manifesto Project
Net migration	Continuous measure of net migration for each country in the corresponding year of survey, retrieved from Eurostat
Unemployment	Continuous measure of the total percentage of unemployed labour force in each country at any given survey year, retrieved from OECD´s Labor Market Statistics (data for Switzerland retrieved from Switzerland in the years 2002-2008 come from Harmonized Unemployment Rate: All Persons for Switzerland
% of university educated	Continuous measure of the percentage of university educated individuals within each country-period calculated directly from the ESS

Table A1.2 Descriptive statistics (mean, standard deviation, minimum and maximum) of variables

Variable	Mean	SD	Min	Max
Attitudes to immigration	16.706	6.19	0	30
(*N*)	(185 154)			
**Independent variables**				
Individual-level				
Age	47.94	16.57	18	85
Lower secondary education	0.16	0.37	0	1
Upper secondary education	0.36	0.48	0	1
University without degree	0.11	0.31	0	1
University degree	0.26	0.44	0	1
Female	0.51	0.49	0	1
Urban residence	0.29	0.45	0	1
Income difficulties	0.14	0.34	0	1
Minority member	0.21	0.40	0	1
Lower-grade service class	0.20	0.40	0	1
Small business owners	0.10	0.31	0	1
Skilled workers	0.31	0.46	0	1
Unskilled workers	0.17	0.37	0	1
unemployed	0.06	0.23	0	1
Left-right scale	5.07	2.05	0	10
Country-Cohort level				
Political climate of equality	5.23	2.62	0.48	16.81
Political climate of tradition	1.55	1.16	0	8.48
% of university educated	34.41	9.23	14.10	54.04
Net migration	1.22	3.25	-14.5	9.9
Unemployment rate	4.87	3.85	0.003	16.55
Country-Period level				
Political climate of equality	4.90	2.12	0.95	9.49
Political climate of tradition	1.66	1.20	0.01	5.69
Net migration	4.23	3.96	-5.6	22.2
Unemployment	6.67	2.62	2.73	15.51
% of university educated	26.86	8.81	0	48.24
(*N*)	(193 224)			

Appendix 2. Historical evolution of the presence of the principles of equality and tradition across cohorts in the countries included

Figure A2.1 Weighted average value of equality across cohorts in the countries included

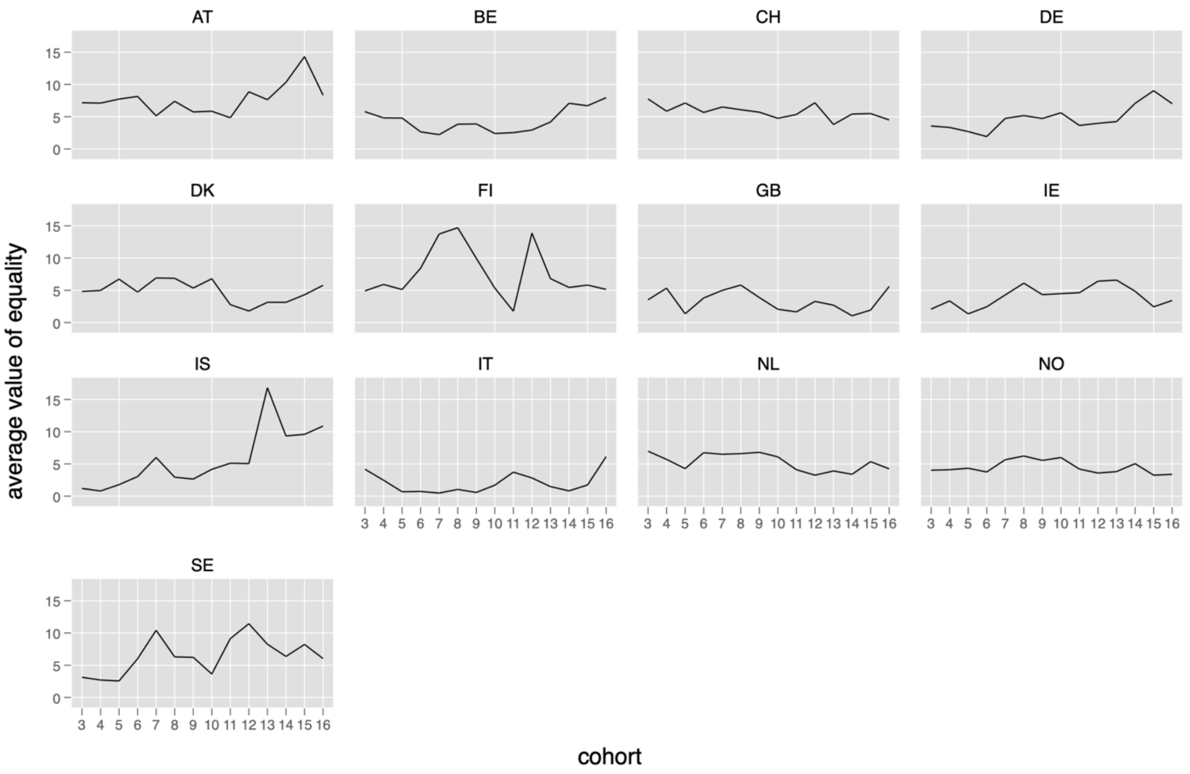

Figure A2.2 Weighted average value of traditionalism across cohorts in the countries included

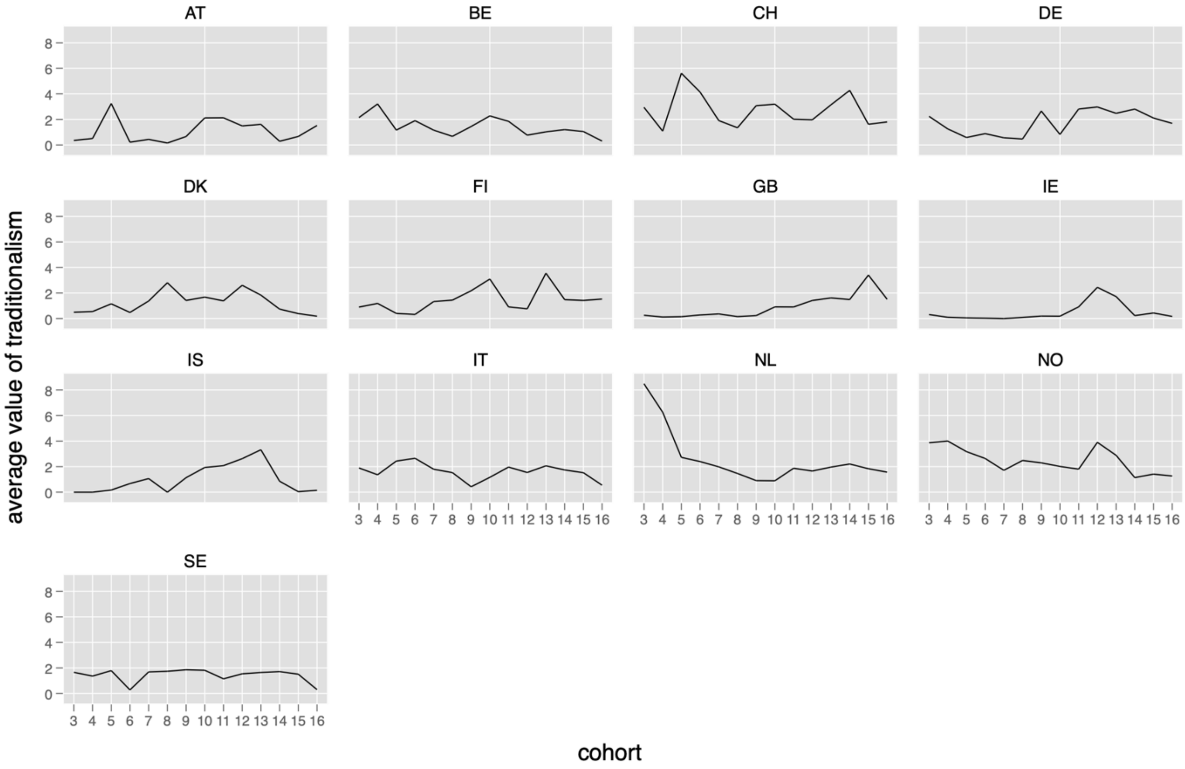

Appendix 3. Robustness of the results when using a different dependent variable (income redistribution)

Table 3.1 Replication of main results when the dependent variable is income redistribution

	Coeff.	S.E.
Intercept	2.480***	(0.0885)
Individual-level		
Age	-0.00510***	(0.000481)
Lower secondary education	-0.00279	(0.0118)
Upper secondary education	0.0546***	(0.0112)
University without degree	0.113***	(0.0130)
University degree	0.168***	(0.0122)
Female	-0.120***	(0.00505)
Urban residence	0.0235***	(0.00555)
Income difficulties	-0.223***	(0.00787)
Minority member	0.0357***	(0.00674)
Unemployed	-0.0644***	(0.0112)
Left-right scale	0.126***	(0.00125)
Lower-grade service class	-0.138***	(0.00814)
Small business owners	-0.0413***	(0.0101)
Skilled workers	-0.219***	(0.00838)
Unskilled workers	-0.245***	(0.00980)
Country-Cohort level		
Political climate of equality	-0.00127	(0.00273)
Political climate of tradition	-0.0147*	(0.00585)
% of university educated	-0.00270***	(0.000752)
Net migration	0.00407+	(0.00246)
Unemployment rate	0.00874**	(0.00286)
Country-Period level		
Political climate of equality	-0.00200**	(0.000617)
Political climate of tradition	-0.000138	(0.000660)
Net migration	-1.23e-06***	(2.08e-07)
Unemployment	-0.0131*	(0.00620)
% of university educated	-0.00208	(0.00171)
Random effect estimates		
Country	0.071	(0.028)
Cohort	0.004	(0.001)
Period	0.007	(0.001)
Individual	0.937	(0.003)
Observations	152 248	

Entries are unstandardized coefficients and standard errors. *** p<0.001, ** p<0.01, * p<0.05

Appendix 4. Robustness of the results when inserting measures of libertarian-authoritarian values

Table 4.1 Replication of main results when controlling for attitudes towards homosexuals at the individual level

	Coeff.	S.E.
Intercept	16.21***	(0.521)
Individual-level		
Age	-0.0115***	(0.0024)
Lower secondary education	0.626***	(0.0656)
Upper secondary education	1.399***	(0.0626)
University without degree	2.204***	(0.0727)
University degree	3.458***	(0.0683)
Female	-0.243***	(0.0282)
Urban residence	0.634***	(0.0308)
Income difficulties	-1.126***	(0.0438)
Minority member	1.598***	(0.0378)
Unemployed	-0.230***	(0.0621)
Left-right scale	-0.440***	(0.0070)
Lower-grade service class	-0.640***	(0.0450)
Small business owners	-0.885***	(0.0562)
Skilled workers	-1.524***	(0.0465)
Unskilled workers	-1.618***	(0.0544)
Homosexuals free to live	-0.971***	(0.0159)
Country-Cohort level		
Political climate of equality	0.0322*	(0.0131)
Political climate of tradition	-0.0318	(0.0280)
% of university educated	-0.0138***	(0.0037)
Net migration	-0.0329**	(0.0119)
Unemployment rate	-0.0372**	(0.0138)
Country-Period level		
Political climate of equality	0.00881+	(0.0045)
Political climate of tradition	-0.00820+	(0.00486)
Net migration	-1.91e-07	(1.49e-06)
Unemployment	-0.197***	(0.0453)
% of university educated	-0.0142	(0.0124)
Random effect estimates		
Country	1.951	(0.814)
Cohort	0.092	(0.015)
Period	0.429	(0.067)
Individual	28.078	(0.103)
Observations	147 866	

Entries are unstandardized coefficients and standard errors. *** p<0.001, ** p<0.01, * p<0.05

Appendix 5. Robustness of the results with a different specification model

Table 5.1 Replication of main results with an alternative model specification (OLS regression)

	Coeff.	S.E.
Intercept	16.21***	(0.521)
Individual-level		
Married	0.129**	(0.0393)
Lower secondary education	0.918***	(0.0762)
Upper secondary education	1.729***	(0.0717)
University without degree	2.630***	(0.0812)
University degree	3.966***	(0.0786)
Female	-0.0589+	(0.0329)
Urban residence	0.734***	(0.0361)
Income difficulties	-1.349***	(0.0511)
Minority member	1.547***	(0.0423)
Unemployed	-0.277***	(0.0712)
Left-right scale	-0.503***	(0.0080)
Lower-grade service class	-0.718***	(0.0523)
Small business owners	-1.019***	(0.0649)
Skilled workers	-1.639***	(0.0541)
Unskilled workers	-1.758***	(0.0632)
Country-Cohort level		
Political climate of equality	0.0306***	(0.0079)
Political climate of tradition	-0.0435*	(0.0174)
% of university educated	-0.00714**	(0.0025)
Net migration	0.00887	(0.0061)
Unemployment rate	0.00421	(0.0067)
Country-Period level		
Political climate of equality	0.0126***	(0.0012)
Political climate of tradition	-0.0144***	(0.0013)
Net migration	1.67e-06**	(5.35e-07)
Unemployment	-0.247***	(0.0130)
% of university educated	-0.0447***	(0.0037)
Observations	112 895	
R-squared	0.211	

Entries are unstandardized coefficients and standard errors. *** p<0.001, ** p<0.01, * p<0.05
